# Neighborhood Physical Disinvestment and Incident Diabetes between visits 1 and 2 of the Hispanic Community Health Study/Study of Latinos (HCHS/SOL)

**DOI:** 10.1007/s11524-026-01061-7

**Published:** 2026-03-16

**Authors:** Cara M. Smith, Elizabeth W. Spalt, Linda C. Gallo, Jordan A. Carlson, Matthew Allison, Bharat Thyagarajan, Earle C. Chambers, Amber Pirzada, Martha Daviglus, Christina Cordero, Qibin Qi, Elena Austin, Amanda M. Fretts, India Ornelas, Robert Kaplan, Joel D. Kaufman, Stephen J. Mooney

**Affiliations:** 1 Department of Environmental and Occupational Health Sciences, School of Public Health, University of Washington, 4225 Roosevelt Way, Office 303J, Seattle, WA 98105 USA; 2 Department of Psychology, San Diego State University, San Diego, CA USA; 3 Department of Pediatrics, Children’s Mercy Hospital and University of Missouri, Kansas City, MO USA; 4 Department of Family Medicine, University of California San Diego, La Jolla, CA USA; 5 Department of Laboratory Medicine and Pathology, University of Minnesota, Minneapolis, MN USA; 6 Department of Family and Social Medicine, Albert Einstein College of Medicine, Bronx, NY USA; 7 Institute for Minority Health Research, University of Illinois at Chicago, Chicago, IL USA; 8 Department of Psychology and Behavioral Medicine Research Center, University of Miami, Miami, FL USA; 9 Department of Epidemiology and Population Health, Albert Einstein College of Medicine, Bronx, NY USA; 10 Department of Epidemiology, School of Public Health, University of Washington, Seattle, WA USA; 11 Department of Health Systems and Population Health, School of Public Health, University of Washington, Seattle, WA USA

**Keywords:** Diabetes, Neighborhood physical disinvestment, Neighborhood environment, Hispanic Community Health Study/Study of Latinos

## Abstract

Neighborhood-scale environmental factors, including disinvestment in infrastructure, may impact cardiometabolic disease risk. To our knowledge, no prior studies have investigated the association between neighborhood disinvestment and incident diabetes. We used a virtual street audit of Google Street View Imagery to generate a neighborhood disinvestment score for participants’ residential addresses in the Hispanic Community Health Study/Study of Latinos (HCHS/SOL). An item response theory model was fit to indicators (litter, graffiti, under-maintained buildings, bars on windows, and abandoned buildings) to form a scale measuring a latent level of disinvestment. Ordinary kriging was used to estimate levels for each residential address within the HCHS/SOL census tracts via spatial interpolation. HCHS/SOL is a longitudinal cohort study of self-identified Hispanic/Latino adults in the Bronx, Chicago, Miami, and San Diego. Using covariate-adjusted and survey-weighted Poisson regression models with data from 9120 participants free of diabetes at baseline (2008–2011), we investigated the association between neighborhood disinvestment and incident diabetes at visit 2 (2014–2017). A sensitivity analysis included only those who did not move during the follow-up period. A one-standard deviation increase in neighborhood disinvestment score was associated with a 13% (95% CI, 1–23%) lower risk of incident diabetes when adjusting for age, sex, education, income, study center/heritage, years in the US, family history, and a neighborhood socioeconomic index. Our sensitivity analysis yielded qualitatively similar results with lower precision. Overall, our analysis does not support the hypothesis that neighborhood physical disinvestment is associated with incident type 2 diabetes in this Hispanic/Latino population.

**Supplementary Information:**

The online version contains supplementary material available at 10.1007/s11524-026-01061-7.

## Introduction

In the United States, around 1.1 million people aged 18 years and older were diagnosed with type 2 diabetes (T2D) in 2021 [1]. Risk of T2D is higher among Hispanic/Latinos, who were found to be 60% more likely to be diagnosed in 2022 compared to non-Hispanic Whites [2]. Well-established T2D individual risk factors include genetics, age, adiposity, diet, and physical activity [3, 4]. Modifiable environmental and neighborhood conditions may also contribute to T2D risk [3, 4], and addressing neighborhood-level risk factors in diabetes prevention interventions may increase effectiveness [5]. The neighborhood built environment is a research area where there are various measures to investigate and various methods and approaches to quantify said measures. The majority of previous research has focused on cross-sectional analysis of disease prevalence, and systematic reviews have highlighted the need for more longitudinal analysis on disease incidence to better investigate a causal relationship [6–8]. Specifically, there is a lack of research on neighborhood physical disinvestment and incidence of diabetes, as related work has primarily focused on cross-sectional analysis of T2D prevalence [9–11].

As diabetes prevalence is higher in low-income urban areas compared to high-income urban areas [5, 12], aspects of the built environment in those areas may contribute to increased risk for diabetes. Previous studies on the built environment have investigated how access to healthy food options and green space [13, 14] is associated with lower T2D risk. In addition to healthy food options and green space, neighborhood disinvestment, a social and economic process in which resources are redirected away from neighborhoods of lower-income populations, may also contribute to T2D risk [5]. Disinvestment is reflected in visual indicators of deterioration and neglect such as deteriorated sidewalks, lack of street lighting, litter, and abandoned buildings. Living within disinvested neighborhoods may increase stress and reduce residents’ ability and desire to engage in physical activity, which may in turn increase diabetes risk [15–17]. Limited physical activity due to the built environment is a modifiable risk factor for obesity and T2D [17–19]. The built environment affecting physical activity levels is of particular concern for older residents, as they may be more affected due to limited physical agility and mobility [16, 20, 21]. Psychological stress is another potential mediator in the relationship between disinvestment and T2D, as experiencing stress from the built environment can lead to physiological and metabolic imbalances, increasing the risk of T2D [22].

Understanding the impacts of living in a disinvested place can be challenging as it can be difficult to operationalize this construct in a tangible and quantifiable way. While theory suggests sociopolitical disinvestment processes degrade diabetes-preventing mechanisms such as economic access to healthy foods and green space quality, these specific features can be challenging to measure directly. Another approach to measuring disinvestment includes gathering data on visual indicators of neighborhood disinvestment via questionnaires [9, 23] or street audits [11].

While prior work has not directly investigated the association between visual indicators of neighborhood physical disinvestment and T2D incidence, perceived neighborhood disorder—a related construct promoted by disinvestment and reflected in vandalism, trash, vacant buildings, and reports of feeling unsafe walking alone—was associated with T2D prevalence in a non-Hispanic White sample of the Health and Retirement Study cohort (OR, 1.11; 95% CI, 1.05, 1.17) [9]. When investigating the interaction between neighborhood disorder and genome-wide genetic risk in the previously mentioned non-Hispanic White sample of the Health and Retirement Study cohort, neighborhood disorder was found to heighten the genetic risk of T2D (OR, 1.10; 95% CI, 1.04, 1.16) [9]. However, a Jamaican cross-sectional survey found no association between neighborhood disorder and diabetes prevalence (OR, 0.99; 95% CI, 0.95, 1.03) [11].

These previous studies on neighborhood disorder and T2D have collected data from study participants via questionnaires [9, 23] or in-person assessment by interviewers [11]. Collecting self-reported exposure and outcome data can lead to “same-source bias” as the participants’ experience with the outcome may influence how they report their exposure. Those experiencing adverse outcomes may be more likely to notice and report potential exposures due to their beliefs and biases, resulting in spurious associations. Approaches to collecting neighborhood environment data to get around the issue of “same-source bias” include asking non-participants living in the same area for their thoughts and opinions [24], collecting data from publicly available databases [12], or conducting in-person street audits [16] and assessments by researchers [11]. A less costly and less time-consuming alternative to in-person street audits is virtual street audits [25, 26]. Visual indicators of neighborhood disinvestment, such as litter and graffiti, are visible on Google Street View and can be combined to create a disinvestment score [25–27]. Utilizing Google Street View increases the spatial availability of visual indicators and allows for comparison across space. Virtual street audits are also a less costly and less time-consuming alternative to in-person street audits [25, 26].

To investigate the relationship between an objective visual measure of the process of neighborhood physical disinvestment and T2D incidence, a virtual audit was conducted using Google Street View (Alphabet, Mountain View, CA) in the neighborhoods of HCHS/SOL study participants. The virtual audit was used to create a neighborhood physical disinvestment score at each participant’s residential address. We used these neighborhood disinvestment scores to investigate the association between indicators of the process of neighborhood disinvestment and incident diabetes between the first and second visits of the HCHS/SOL.

## Methods

### Study Design and Population

HCHS/SOL is a longitudinal cohort of 16,415 self-identifying Hispanic/Latino adults who were at least 18 years of age at enrollment and were recruited from the Bronx, Chicago, Miami, and San Diego [28]. More details on the HCHS/SOL sample and study design have been previously reported [29, 30]. Briefly, the first in-person visit and enrollment occurred between 2008 and 2011 in each of the four cities. Yearly follow-up phone calls were conducted, and a second round of in-person visits (visit 2) occurred between 2014 and 2017.

Questionnaires and clinical examinations were administered at both in-person visits. The questionnaires assessed demographics, current health and medical history, socioeconomic status, and residential information [30]. Clinical examinations included the collection of blood and urine for various assays and analyses [30]. The yearly follow-up phone calls contained questions on general health, potential updates from their doctors or health professionals, and details on any potential hospitalization or emergency room visits that occurred since the last follow-up, as well as any updates to their residential location [30]. The study population for this analysis included participants who completed the baseline and second visits, were free of diabetes at the baseline visit, and had geocoded residential address data at both visits. All participating institutions received approval from their respective institutional review boards, and written informed consent was received from all participants.

### Diabetes Ascertainment

Ascertainment of diabetes occurred at baseline, during the yearly follow-up phone calls, and at visit 2. During calls and visits, participants were asked if a doctor or health care professional had told them they had diabetes or high sugar in their blood and if they were receiving any treatment [30]. However, only during the in-person visits was blood collected and assayed to measure fasting plasma glucose (FPG) and glycosylated hemoglobin (HbA1c) levels [30]. A 2-h oral glucose tolerance test (OGTT) was also conducted on participants who did not self-report a diabetes diagnosis and on participants with FPG < 150 mg/dL [31]. Ascertainment of diabetes in the HCHS/SOL cohort was not able to distinguish between type 1 and type 2 diabetes.

For this analysis, we use two definitions of diabetes. Each definition is based on the American Diabetes Association criteria [32] and on data collection practices. Our primary definition for diabetes was having ascertainment of diabetes at visit 2 and any of the following: an FPG ≥ 126 mg/dL; an HbA1c level ≥ 6.5%; a post-OGTT glucose ≥ 200 mg/dL; or use of antihyperglycemic drugs. Our secondary definition, while also limited to those with ascertainment of diabetes at visit 2, included those who fit the primary definition of T2D and those who self-reported diabetes or high sugar in their blood during the in-person visits or the annual follow-up phone calls.

Pre-diabetes was assessed at baseline and at visit 2. Based on the American Diabetes Association criteria, pre-diabetes was defined as having an FPG ≥ 100 mg/dL and < 126 mg/dL and an HbA1c level ≥ 5.7% and < 6.5% [32].

### Google Street View Audit

In order to generate an interpolated disinvestment measure for each residential address, we conducted a virtual street audit using an established, validated approach and Google Street View [25, 26]. For each of the four cities, we selected 1000 street locations to be virtually audited via Google Street View. Locations were selected within the 351 census tracts where participants lived at baseline using a 600-point grid. To increase precision in neighborhoods where most participants lived, we embedded an additional 600-point oversample in the census tracts with the top quintile of HCHS/SOL participants in each study center. All points were randomly jittered up to 250 m. We used the Computer-Assisted Neighborhood Visual Assessment System (CANVAS) to conduct the virtual street audit [33]. We uploaded our set of coordinates to CANVAS, which tried to match each coordinate to Google Street View imagery within 50 m. If there were no matches within 50 m, the location was not used. For each location, auditors answered a set of 53 questions related to neighborhood disinvestment based on the most recent image (range, 2007–2023; median, 2022) and the oldest image (range, 2007–2019; median, 2011) using the drop and spin method (Table S1). The drop and spin method refers to how CANVAS accessed Google Street View at each location, and auditors were only able to spin around to answer the questions. They were not able to “walk” up and down the street, as this could change the date of the images.

Audit questions were taken from previously validated audits designed to assess neighborhood disinvestment and pedestrian safety [26, 34, 35]. Examples of the questions auditors had to answer were “Is there garbage, litter, or broken glass in the street or on the sidewalk?,” “Are there abandoned cars?,” and “Do you see boarded up or abandoned buildings?” The full list of questions is available in the supplementary material (Table S1). All responses were coded categorically, and most were dichotomous. The virtual street audit was conducted by 12 undergraduate and graduate students at the University of Washington between July 2022 and May 2023. All auditors were trained on the same set of example locations. During training, all questions and examples were discussed to limit subjectivity among auditors. For example, multiple images of graffiti and murals were shown during training to highlight the differences between them. To assess agreement between the auditors, approximately 15% of the locations in each study center were randomly selected as a reliability subsample. Auditor training materials are available upon request from the corresponding author.

### Neighborhood Disinvestment

To generate a neighborhood disinvestment measure for each participant’s residential address, we used a validated approach involving an item response theory (IRT) model and ordinary kriging [25, 26]. Only data from the most recent or only image available at a location were used to fit the IRT model. We considered creating a spatiotemporal disinvestment model with data from the oldest and most recent images; however, we found there was too little change over time to warrant a model [36]. We used the most recent, or only, images instead of the oldest images because the oldest images were less spatially dense. An IRT model was fit to indicators (litter, graffiti, under-maintained buildings, bars on windows, and abandoned buildings) to form a scale measuring a latent level of disinvestment. The IRT model’s posterior probability of observing the indicators we actually observed was used to estimate a latent level of disinvestment at each audited location. We then used ordinary kriging to estimate levels for each residential address within the HCHS/SOL census tracts via spatial interpolation. Geocoded addresses with the disinvestment measure were then matched with participants’ geocoded addresses in a secure research workspace. Our neighborhood disinvestment measure is unitless, with higher values indicating more disinvestment. For this analysis, we have transformed the measure to its *z*-score, indicating a unit increase as a change in 1 standard deviation. More information on how the neighborhood disinvestment measure was developed is presented in the supplementary material.

### Inclusion Criteria

Of the 16,415 HCHS/SOL study participants, ascertainment of diabetes was collected at visit 2 for 11,619 participants. Of the 11,619 participants, we had a neighborhood disinvestment measure for 11,503 at their baseline residential address. For our primary analysis, participants needed to be free of diabetes at baseline based on the primary diabetes definition (*N* = 9120). For our analysis with the secondary diabetes definition, 8989 were free of diabetes at baseline.

### Statistical Analysis

To compare the two definitions of diabetes, we computed demographic statistics for both samples and calculated an age-centered adjusted incidence rate. Range, median, mean, and standard deviation of neighborhood disinvestment were also calculated for each sample overall and stratified by study center. We ran Pearson’s correlations on disinvestment at baseline and a neighborhood socioeconomic status (NSES) index [37], population within a 1 km buffer, and percent Hispanic at the census tract level. We ran Spearman’s rank correlations between disinvestment and ordinal data on individual-level education, income, and years living in the US. The NSES index is a compositional measure of NSES at the census tract level that represents education, employment, housing, income/wealth, occupation, and residential stability [37]. Higher index scores indicate higher NSES disadvantage [37].

For our primary analysis, we investigated the association between neighborhood disinvestment at baseline residential addresses and incident diabetes at visit 2 using the primary diabetes definition. We ran Poisson regression models using a log link and years between baseline and visit 2 as the offset for follow-up time to generate an incidence rate ratio (IRR) comparing groups with a one-standard deviation difference in neighborhood disinvestment. All regression models used complete-case analyses and sampling weights to account for the HCHS/SOL complex sampling design, those living in the same household, and participant non-response at visit 2, using the *survey* package in R [29]. The sampling weights were also calibrated to age, sex, and Hispanic/Latino heritage from the 2010 US Census.

Minimally adjusted models were adjusted only for age and sex. Our fully adjusted model was adjusted for age, sex, years living in the USA (born in the 50 USA states, foreign born in the USA for more than 10 years, or foreign born in the USA for less than 10 years), a 5-level income variable, a 4-level educational attainment variable, family history of diabetes, an NSES index [37], and a 17-level variable combining study center and Hispanic/Latino heritage group. Study center-specific models were created using the same sets of covariates, except that the 17-level combination variable was replaced with the 8-level Hispanic/Latino heritage variable (Cuban, Dominican, Puerto Rican, Mexican, Central or South American, more than one heritage, or other).

We repeated the regression models to investigate the association between neighborhood disinvestment and three different progressions of diabetes: (1) progression from pre-diabetes to diabetes, where the sample only included those with pre-diabetes at baseline; (2) progression of those free of diabetes and pre-diabetes to diabetes; and (3) progression of those free of diabetes and pre-diabetes at baseline to pre-diabetes at visit 2. Results from all models were exponentiated to report the IRR for each unit increment in neighborhood disinvestment.

### Sensitivity Analyses

We repeated the Poisson regression models using the secondary definition of diabetes, which included a self-reported diagnosis. As 46.4% of participants moved residences during the follow-up period, we also conducted a sensitivity analysis restricted to those who did not move (*N* = 6164).

### Missing Data

For the analytic sample, the only variable with greater than 5% missingness was income, with 7.6% of participants missing data. Due to concerns about missing data and the computational intensity of multiple imputations by chained equations (MICE), we conducted a MICE analysis as the sensitivity analysis in two model iterations. The MICE analysis consisted of 10 imputations and 50 iterations on the fully adjusted model for both the primary and secondary definitions of diabetes. Results from the MICE analysis were very similar and did not change the overall interpretation of results. Since completing MICE analyses on all our models would be computationally intense, and the MICE analysis tested in our two iterations yielded very similar results, we used complete-case analysis for our reported analyses.

All analyses were conducted in R Studio, with R version 4.1.3 (R Foundation for Statistical Computing, Vienna, Austria). The “ltm” package (version 1.2) was used for the IRT models. The “mice” package (version 3.15.0) was used for the MICE analysis [38], and the “survey” package (version 4.2–1) was used for incorporating the survey design and weights into the regression models [39].

## Results

The analytic sample comprised 9120 participants who were free of diabetes under our primary definition of diabetes (excluding self-reported diagnosis) at baseline. There were 1038 incident cases at visit 2, with an age-centered adjusted incidence rate of 15.9 per 1000 person-years. For our secondary definition of diabetes (including self-reported diagnosis), 8989 participants were free of diabetes at baseline, and there were 1476 incident cases by visit 2, with an age-centered adjusted incidence rate of 23.5 per 1000 person-years. Weighted summary characteristics of participants by diabetes status at visit 2 are presented in Table 1.

**Table 1:** Weighted summary characteristics of participants by diabetes status at visit 2 using primary definition of diabetes

	Sample weighted % or mean (SD)	
**Variables**	Diabetes at visit 2 (*N* = 1038)	Free of diabetes at visit 2 (*N* = 8082)
**Female**	47.0	50.9
**Age, years**	46.7 (12.8)	38.2 (14.1)
**Hispanic/Latino heritage**		
Central American	4.9	7.5
Cuban	19.7	18.5
Dominican	9.4	9.6
Mexican	39.0	39.5
Puerto Rican	18.9	14.7
South American	3.3	5.5
More than one heritage	4.8	3.6
Other	0.2	1.0
**Years in the US**		
Less than 10 years	23.8	28.5
10 years or more	56.8	46.5
US born	19.4	24.9
**Family history of diabetes**	50.1	36.4
**Education**		
No high school diploma or GED	36.2	27.9
At most a high school diploma or GED	28.2	28.8
High school (or GED) education	12.0	13.1
University/college education	24.7	30.2
**Income**		
Less than $10,000	15.3	13.8
$10, 001–20,000	31.4	31.6
$20, 001–40,000	34.3	33.9
$40, 001–75,000	13.1	15.1
More than $75,000	6.0	5.7
**Neighborhood SES Index**	1.1 (0.8)	1.1 (0.8)
**Years between baseline and visit 2**	6.0 (0.8)	6.1 (0.9)
**Study center**		
Bronx	30.1	28.1
Chicago	16.9	15.8
Miami	27.8	29.7
San Diego	25.3	26.4
**Neighborhood disinvestment**		
Overall	− 1.1 (0.5)	− 1.1 (0.5)
Bronx	− 0.6 (0.3)	− 0.56 (0.3)
Chicago	− 1.3 (0.4)	− 1.2 (0.3)
Miami	− 1.2 (0.4)	− 1.2 (0.4)
San Diego	− 1.5 (0.4)	− 1.5 (0.4)

**Fig. 1:**
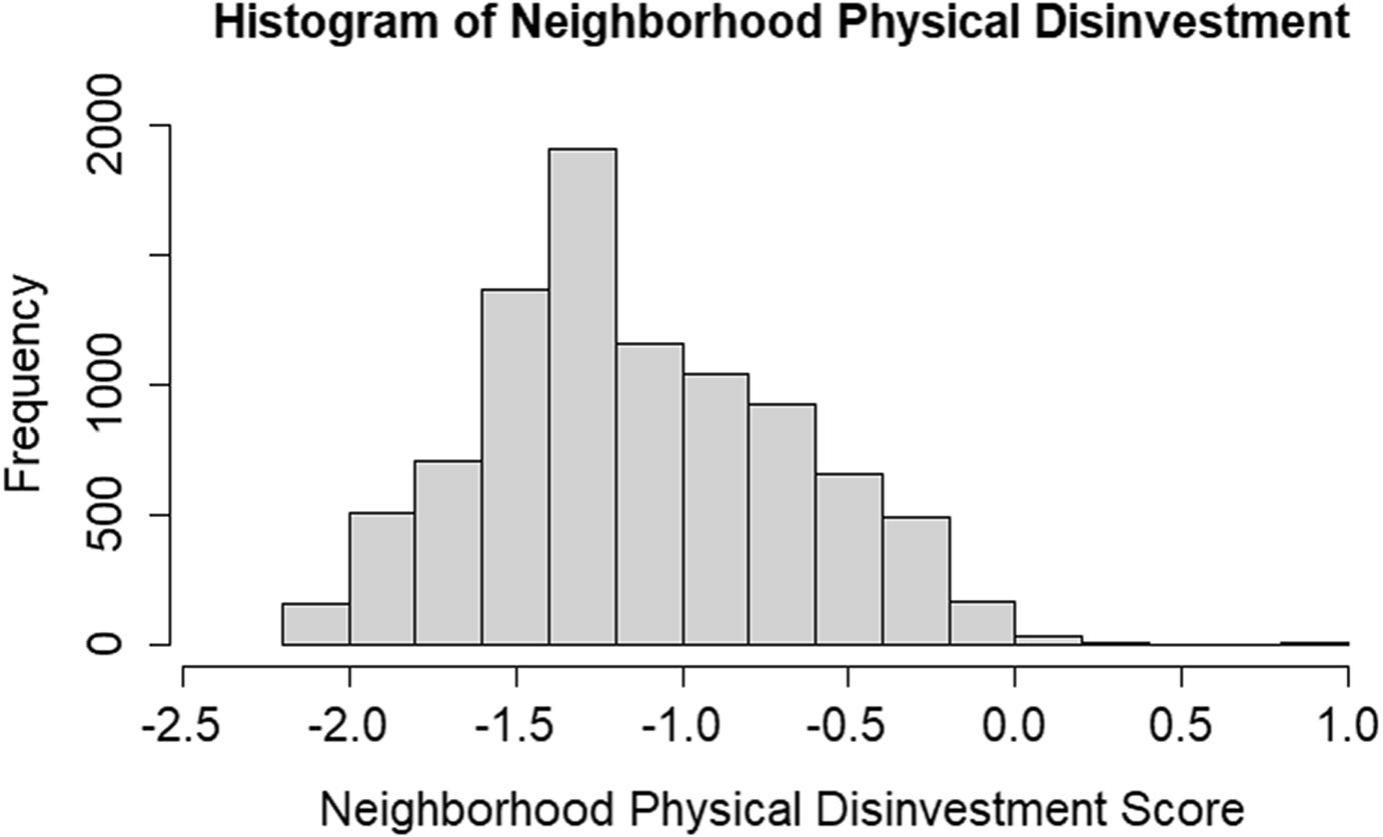
Histogram of the neighborhood physical disinvestment score at the baseline residential addresses of those free of diabetes at baseline

Baseline disinvestment distribution was slightly right-skewed and ranges from − 2.07 to 0.81 with a median of − 1.21 and a mean and standard deviation of − 1.13 and 0.47, respectively. Histogram of the neighborhood physical disinvestment score is presented in Fig. 1. Baseline disinvestment *z*-score ranged from − 2.00 to 4.12. Baseline disinvestment was moderately correlated with NSES (*r* = 0.56) and population within a 1 km buffer (*r* = 0. 64). Baseline disinvestment was not correlated with individual level education ($\rho$ = − 0.07), income ($\rho$ = − 0.18), years in the USA ($\rho$ = 0.04), or percentage of Hispanic/Latino population at the census tract level (*r* = ** − **0.17).

For our primary analysis, utilizing the primary definition of diabetes, each one-standard deviation increase in neighborhood disinvestment was associated with a 13% decreased risk of incident diabetes (IRR = 0.87; 95% CI, 0.77–0.99) in the fully adjusted model (Table 2). Removing the adjustment for NSES yielded very similar results to the fully adjusted model (Table S2). IRR for all but two other model iterations, including stratification by study center, were not significant (Table S3 and S4). Among those free of pre-diabetes and diabetes at baseline, each one-standard deviation increase in neighborhood disinvestment was associated with a 34% decreased risk of incident diabetes (IRR = 0.66; 95% CI, 0.44–0.99) in the fully adjusted model (Table S4). Among those at the Miami study center free of diabetes at baseline, each one-standard deviation increase in neighborhood disinvestment was associated with a 20% decreased risk of incident diabetes (IRR = 0.80; 95% CI, 0.64–0.99) in the fully adjusted model (Table S4).

**Table 2:** Minimally and fully adjusted incidence rate ratios of diabetes (primary and secondary definitions) at visit 2 based on neighborhood disinvestment *z*-score at baseline

	**Minimally adjusted**^**a**^			**Fully adjusted**^**b**^		
**Outcome**	***N***	**Cases at visit 2**	**IRR (95% CI)**	***N***	**Cases at visit 2**	**IRR (95% CI)**
Primary diabetes (lab results and/or self-reported medication)	9120	1038	0.97 (0.89–1.06)	8346	956	0.87 (0.77–0.99)*
Secondary diabetes (primary + self-reported diagnosis)	8989	1476	0.95 (0.88–1.02)	8230	1362	0.95 (0.86–1.06)
Primary diabetes (subset of non-movers)	4727	584	1.05 (0.94–1.17)	4373	544	1.05 (0.90–1.23)

*IRR* incident rate ratio

^*^P < 0.05

^a^Adjusted for age and sex

^b^Adjusted for age, sex, years living in the USA, income, educational attainment, family history of diabetes, NSES, and study center/ethnic heritage

Results from our primary analysis were similar for our sensitivity analysis utilizing the secondary definition of diabetes (Table 2). The sensitivity analysis on the subset of participants who did not move during follow-up yielded similar non-significant results, with IRRs around 1, suggesting no association. For the fully adjusted model, utilizing the primary definition of diabetes, results were in the expected direction but not statistically significant (IRR = 1.05; 95% CI, 0.90–1.23) (Table 2). For the study-center-specific models among those who did not move, the fully adjusted model for Chicago was statistically significant with an IRR of 0.72 (95% CI, 0.56–0.96) (Table S5). All other study centers had a negative association between disinvestment and incidence of diabetes, except for the fully adjusted model for the Bronx (IRR, 1.05; 95% CI, 0.78–1.42) (Table S5).

## Discussion

Using an image-based assessment of built environment conditions for a large urban Hispanic/Latino population, we found evidence of an association between visual indicators of the process of neighborhood disinvestment and lower diabetes incidence in our primary analysis. These results were contrary to our hypothesized association and were not robust to our additional analysis of diabetes progression or to our sensitivity analyses.

Ours is the first study relating an independently observed measure of visual indications of neighborhood physical disinvestment to incident diabetes. Our results are consistent with generally mixed findings of prior studies assessing the association between related aspects of the neighborhood and diabetes or HbA1c levels. For example, a previous study in Jamaica did not find an association between neighborhood disorder, assessed by an in-person audit, and diabetes prevalence (OR, 0.99; 95% CI, 0.95–1.03) [11]. Another study in Jackson, Mississippi found a positive association between a survey-based measure of neighborhood problems (noise, heavy traffic, speeding cars, lack of access to adequate food or shopping, litter) and incident diabetes (HR, 1.24; 95% CI, 1.00–1.54) among African Americans [40]. An analysis conducted at the San Diego site of the HCHS/SOL did not find an association between neighborhood indicators of social disorder, measured by per capita liquor stores, crime rates, vacant households, and vacant land, and change in HbA1c levels between baseline and visit 2 [12].

However, studies exploring neighborhood deprivation and disadvantage have more consistently found indications that disadvantage is associated with diabetes. The previously mentioned analysis at the San Diego site of the HCHS/SOL identified an association between higher neighborhood socioeconomic deprivation (a composite of educational attainment, income level, unemployment, crowded households, female-headed households with children, public health insurance, and households receiving public assistance) and worsening diabetes status at visit 2 (OR = 1.27, 95% CI = 1.10, 1.46) [12]. More broadly, a harmonized analysis of three independent studies found that increasing neighborhood disadvantage, measured by census tract data on income, education, unemployment, poverty, households on public assistance, and households with no cars, was generally associated with an increased risk of T2D [41].

There are several potential explanations for the overall lack of association between neighborhood disinvestment and incident diabetes in our analysis. For one, our neighborhood disinvestment measure had low variability overall (IQR = 0.65) and within each study center (each IQR < 0.60), which may have limited our ability to detect an association in an epidemiologic study. Future studies utilizing this method would benefit from greater variability in neighborhood conditions.

As we hypothesized, neighborhood disinvestment would affect T2D risk via increasing stress and reducing residents’ ability and desirability to engage in physical activity [15–17], another potential explanation for our overall lack of association may be due to unmeasured confounding related to stressors and barriers to physical activity. Physical activity is a well-known protective factor for the development of T2D and antecedents of T2D, including obesity. Individuals living in more disinvested neighborhoods may engage in less physical activity because they feel less safe and may find physical activity to be hazardous [17]. Individuals living in more disinvested neighborhoods may also have less access to resources that support physical activity. In the Multi-Ethnic Study of Atherosclerosis (MESA), long-term exposure to residential environments with greater resources to support physical activity was found to be associated with incident T2D [42]. Given this postulated mechanism, we did not adjust for physical activity as we believe physical activity mediates the disinvestment–T2D relationship. The relationship between disinvestment, physical activity, and T2D should be further investigated in future studies. Future studies should account for where physical activity occurs and how one’s neighborhood environment may influence physical activity choices.

Disinvestment could also be related to other neighborhood factors such as the availability of healthy foods or the support for active transportation; factors which we did not capture with Google Street View images. Given the positive correlation between population density and neighborhood disinvestment, dense neighborhoods may have more walkable destinations, and therefore residents may be more likely to use physically active forms of transportation [43]. Future studies should consider how the food environment, access to recreational spaces, and neighborhood walkability interact with neighborhood disinvestment to influence T2D risk.

Another potential explanation for our results may be related to the mixed effects of living in ethnic enclaves on health. Our measure could potentially reflect the beneficial effects of living in ethnically dense communities. Hispanic/Latino immigrants often settle in neighborhoods that have been established by other Hispanic/Latino residents due to their lower housing costs, a high number of native Spanish speakers, and a perceived safety from law enforcement arising from “blending in” more [44, 45]. Immigrants may find living in these enclaves to be protective and less stressful [46]. Living in neighborhoods with high immigrant populations may protect against obesity based on previous cross-sectional analyses; however, these may be confounded by residential self-selection [47]. An analysis of residential segregation and incidence of metabolic syndrome in the HCHS/SOL cohort yielded mixed results on health effects for those living in areas with a high concentration of Hispanic/Latino residents [48]. Health effects of ethnic enclaves, which tend to have more residential disadvantages than neighborhoods with high non-Hispanic white populations [45], may depend on nativity and how long one has been in the US. While we did adjust for years in the US, interrogating this relationship in HCHS/SOL is challenging due to the clustering of participants with similar backgrounds in small neighborhoods. Future studies need to investigate the structural relationship between neighborhood disinvestment, ethnic enclaves, and T2D in more detail.

### Strengths and Limitations

Our study has notable strengths, including being the first study to investigate the relationship between visual indications of neighborhood disinvestment and incident type 2 diabetes. The HCHS/SOL allowed us to investigate this relationship in a large, diverse population-based cohort of Hispanics/Latinos, an underrepresented group in research, in four major USA cities. The comprehensiveness of the HCHS/SOL dataset allowed us to adjust for important individual-level confounders. Conducting a validated virtual audit system using Google Street View allowed us to generate a disinvestment measure in a quick and cost-effective manner compared to an in-person street audit [25].

Our analysis was not without limitations. First, the baseline occurred between 2008 and 2011; however, the images used to derive the disinvestment variable were taken between July 2007 and April 2023 with most images from 2022, creating a temporal incompatibility. However, based on previous analysis, there was little variation between the oldest and most recent images [36]. Using images from this audit that do not temporally match our time of interest (baseline) is unlikely to bias our results [36]. Second, our Google Street View Imagery sample for the virtual audit averaged 10 locations per census tract. While we believe our sampling approach to be adequate based on previous evidence that sparser sampling does not lead to imprecise estimates [25], there is the possibility that higher heterogeneity within certain census tracts not previously tested may lead to less precise estimates. However, as noted previously, this sampling approach had been validated in different contexts [25, 26]. Additionally, we did not aggregate our disinvestment measure to the census tract level. We used ordinary kriging to estimate levels at each residential address via spatial interpolation. Third, the average length of follow-up was 6 years, which may be too short to see any effects as T2D can take several years to develop. Additionally, the mean age of participants free of diabetes at Visit 2, 38.2 years old, is younger than the mean age of diabetes diagnosis of Mexican-Americans, 44.9, from 2011 to 2018 [49]. We are unable to expand analysis to include years before the baseline visit as we do not have residential data prior to their visit. Fourth, we are unable to distinguish between type 1 and type 2 diabetes in our ascertainment. However, given the age of the HCHS/SOL cohort, the greater majority of diabetes cases are likely to be type 2. Lastly, our neighborhood disinvestment measure was only focused on their residential neighborhood, an environment where participants do not spend all of their time. The neighborhood environment outside their residential area, such as their place of work and larger community environment, was unaccounted for and may affect T2D risk as well.

## Conclusion

In conclusion, our results did not support our hypothesis of an association between visual indications of neighborhood disinvestment and incident diabetes in the HCHS/SOL cohort. Future studies should continue to evaluate the role of the food environment, greenspace, and physical activity in considering neighborhood-scale infrastructure impacts on diabetes risk.

## Supplementary Information

Below is the link to the electronic supplementary material.

ESM 1 (1.34 MB DOCX)
