# Supplementary material for: Neighborhood Physical Disinvestment and Incident Diabetes between visits 1 and 2 of the Hispanic Community Health Study/Study of Latinos (HCHS/SOL)
Source: J Urban Health. 2026 Mar 16;103(2):382–94. doi: 10.1007/s11524-026-01061-7 (PMC13235673; doi:10.1007/s11524-026-01061-7)
Supplement: Supplementary file 1 — (1.34 MB DOCX) [file 11524_2026_1061_MOESM1_ESM.docx]

**Title:** Neighborhood Physical Disinvestment and Incident Diabetes between visits 1 and 2 of the Hispanic Community Health Study/Study of Latinos (HCHS/SOL)

**Supplementary Material**

**Appendix on Disinvestment Measure**

**Overview**

We developed our measure of visual indications of neighborhood disinvestment following the procedure developed by Raudenbush & Sampson (1999), adapted for Google Street View audits by Mooney et al (2014) and further refined by Plascak et al (2020). Roughly, this process has 5 steps:

1. Conduct ‘drop-and-spin’ virtual audits assessing visual indicators of neighborhood disinvestment including graffiti, unrepaired buildings, etc., drawing from the Project for Human Development in Chicago Neighborhoods and Irvine-Minnesota systematic social observation inventories on a spatially dense sample of Google Street View images.
2. Assess inter-rater reliability of individual items, potentially dropping observations from auditors whose responses appear unreliable and/or audit items for which even modest reliability (Kappa > 0.2) could not be established
3. Fit an Item Response Theory model to remaining items, assessing model fit statistics, item discrimination, and range of assessed severity to select a final model. For each audited location, assign the modal posterior latent score as the visual indicators of disinvestment score for that location.
4. Then use the disinvestment scores in audited locations in an ordinary kriging model to spatial interpolate to estimate the visual indicators of disinvestment present at the centroid of each land parcel in each HCHS/SOL city.
5. In the HCHS/SOL secure research workspace, spatially link the disinvestment score to the land parcel containing the study participant’s home address.

**Step 1. Audit protocol and candidate indicators**

The following items were used in virtual audits. Each item was assessed on the oldest and most recent image.

| Question posed to auditors | Responses indicating disinvestment | Responses not indicating disinvestment |
| --- | --- | --- |
| Is there vacant or undeveloped land? | Yes | No |
| Do you see any windows with bars on them? | Yes | No; Not applicable |
| Is there garbage, litter, or broken glass in the street or on the sidewalks? | Yes | No |
| Are there empty beer or liquor bottles visible in streets, yards, or alleys? | Yes | No |
| Are there abandoned cars? | Yes | No |
| Is there graffiti, or evidence of graffiti that has been painted over, on buildings, signs, or walls? | Yes | No |
| How many buildings, driveways, or other private infrastructure need non-trivial repairs? | One; Two or more | None; Not applicable |
| Do you see burned out buildings? | Yes | No |
| Do you see boarded up or abandoned buildings? | Yes | No |

**Step 2. Inter-rater reliability**

Four hundred and twenty-seven locations were randomly selected for an inter-rater reliability subsample. Answers where a rater indicated they could not tell or skipped over a question were treated as missing. No auditors’ observations were dropped due to observable patterns of systematic errors

| Audit Item | Kappa |
| --- | --- |
| Buildings Needing Repairs | 0.22 |
| Boarded Up Buildings | 0.24 |
| Vacant Lot | 0.27 |
| Empty Bottles | 0.31 |
| Litter | 0.43 |
| Burned Out Buildings | 0.44 |
| Abandoned Cars | 0.55 |
| Graffiti | 0.64 |
| Window Bars | 0.71 |

**Step 3. Item Response Theory scale development**

After exploring item frequency across the 4 HCHS/SOL cities and visual observation of item characteristic curves, we selected a five-item scale comprising litter, graffiti, bars on windows, abandoned buildings and presence of buildings needing repairs. Item characteristic curves and estimated disinvestment scores by city are shown below


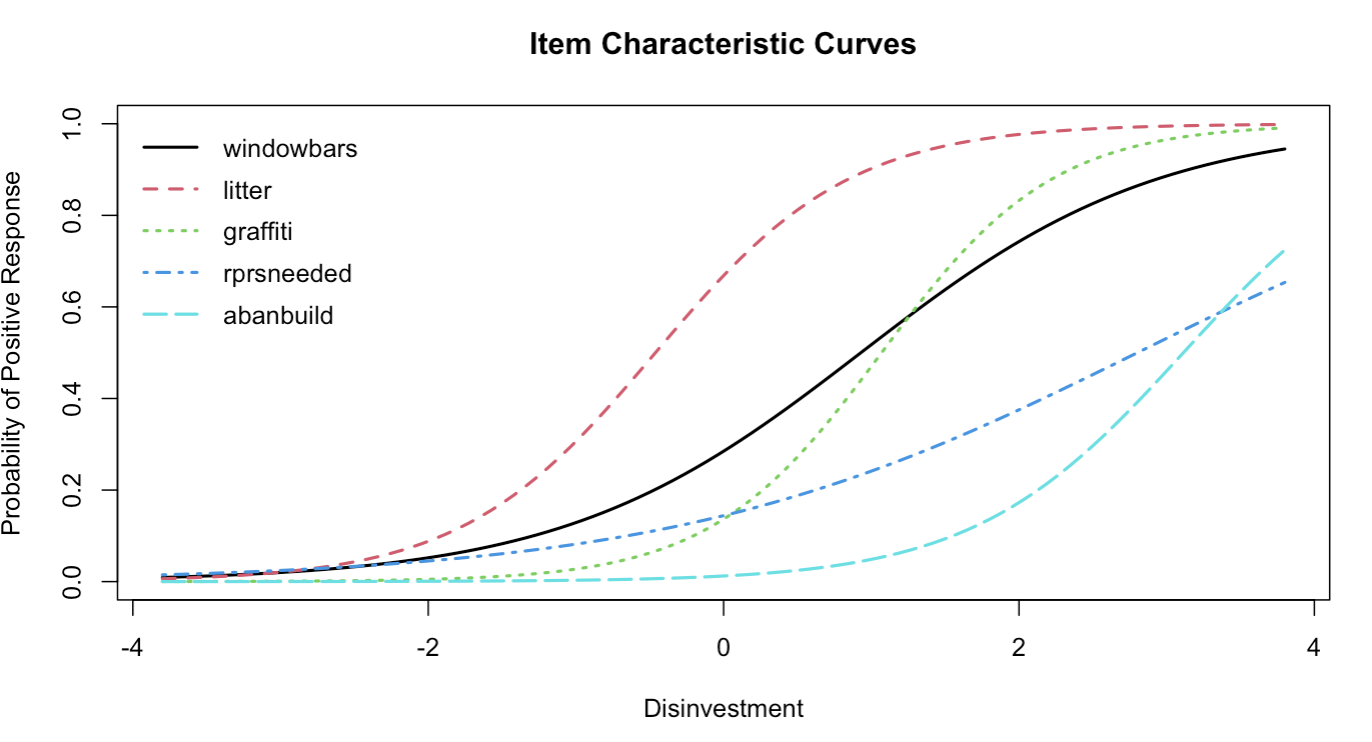


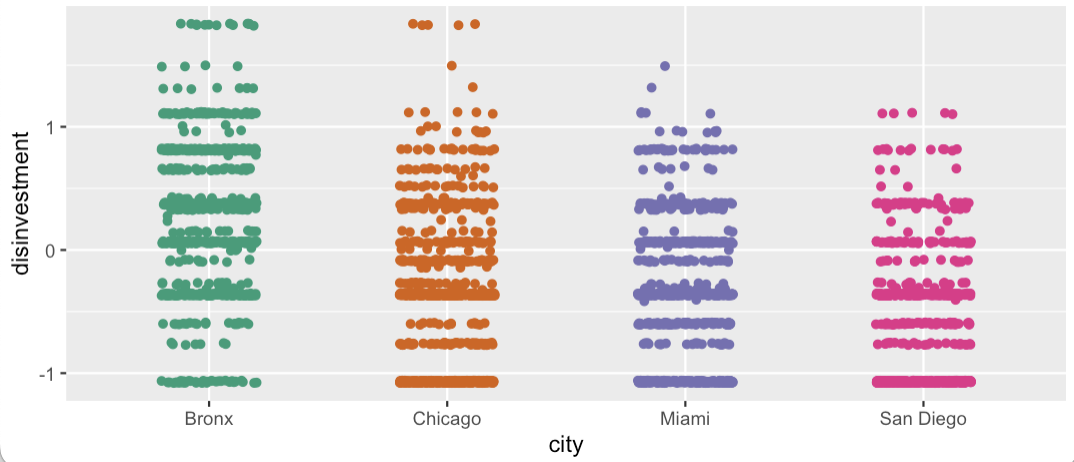


**Step 4. Spatial interpolation**

Visual inspection of within-city variograms confirmed a pattern of spatial autocorrelation consistent with the use of ordinary kriging to spatially interpolate to the parcel level. For example, the upward curve of the variogram for the Bronx


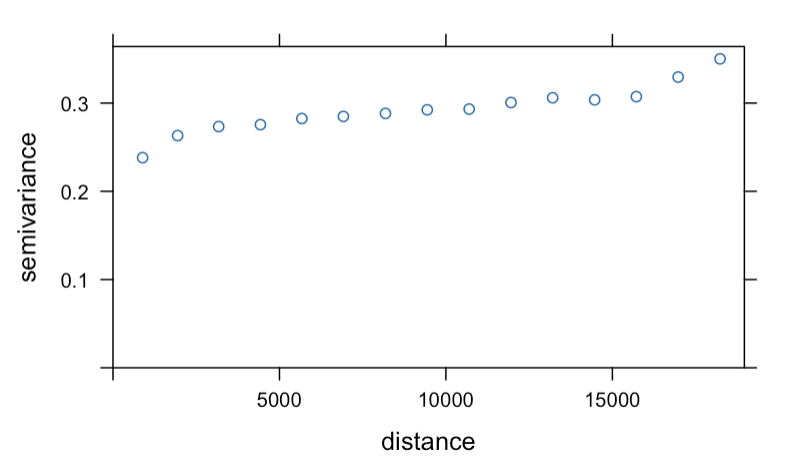


Confirms that nearby observations predict disinvestment better than more remote observations. The corresponding map of physical disinvestment in the Bronx


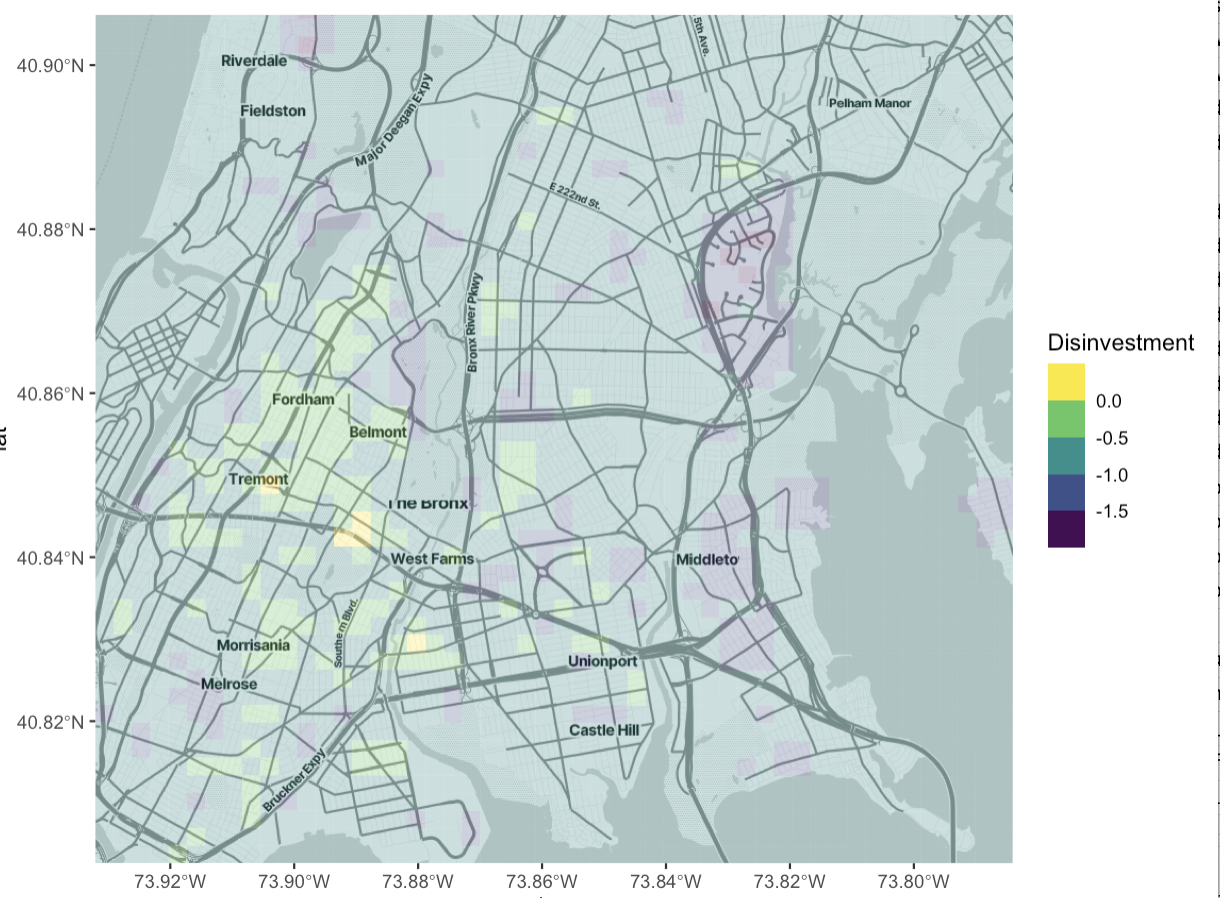


Shows higher level of disinvestment near the West Farms/Crotona Park area of the central Bronx and lower levels in Co-op City in the northeast Bronx, consistent with face value expectations for spatial variability in disinvestment in the Bronx.

**Step 5. Link to HCHS/SOL participants**

Using parcel-level spatial linkage, we were able to link 16,177 (98.6%) participant baseline addresses disinvestment measures. We were unable to match with 100% of participants as there were 175 participants without geocoded baseline addresses and there were 63 participants with baseline addresses more than 250 m from any land parcel with a disinvestment measure.

**References**

Raudenbush, S.W. and Sampson, R.J., 1999. Ecometrics: toward a science of assessing ecological settings, with application to the systematic social observation of neighborhoods. *Sociological methodology*, *29*(1), pp.1-41.

Mooney, S.J., Bader, M.D., Lovasi, G.S., Neckerman, K.M., Teitler, J.O. and Rundle, A.G., 2014. Validity of an ecometric neighborhood physical disorder measure constructed by virtual street audit. *American journal of epidemiology*, *180*(6), pp.626-635.

Plascak, J.J., Rundle, A.G., Babel, R.A., Llanos, A.A., LaBelle, C.M., Stroup, A.M. and Mooney, S.J., 2020. Drop-and-spin virtual neighborhood auditing: assessing built environment for linkage to health studies. *American journal of preventive medicine*, *58*(1), pp.152-160.

**Supplementary Tables**

**Table S1**. List of questions auditors answered for each location and the answer options.

| Question | Answer Options |
| --- | --- |
| 1) Is this view appropriate for auditing | Yes/No/Cannot Tell |
| 2) Is there vacant or undeveloped land?* | Yes/No/Cannot Tell |
| 3) Do you see any windows with bars on them?* | Yes/No/Cannot Tell |
| 4) Is there garbage, litter, or broken glass in the street or on the sidewalks? * | Yes/No/Cannot Tell |
| 5) Are there empty beer or liquor bottles visible in streets, yards, or alleys?* | Yes/No/Cannot Tell |
| 6) Are there abandoned cars?* | Yes/No/Cannot Tell |
| 7) Is there graffiti, or evidence of graffiti that has been painted over, on buildings, signs, or walls?* | Yes/No/Cannot Tell |
| 8) How many buildings, driveways, or other private infrastructure need non-trivial repairs?* | None/One/Two or more/Not applicable/ Cannot Tell |
| 9) Do you see burned out buildings?* | Yes/No/Cannot Tell |
| 10) Do you see boarded up or abandoned buildings?* | Yes/No/Cannot Tell |
| 11) Is there a sidewalk? | Yes/No/Cannot Tell |
| 12) Is the sidewalk continuous? | Yes/No/Cannot Tell |
| 13) Are there major bumps, cracks, holes, or weeds in the sidewalk? | Yes/No/Cannot Tell |
| 14) Is the sidewalk unobstructed? | Yes/No/Cannot Tell |
| 15) Should there be a sidewalk here? | Yes/No/Cannot Tell |
| 16) Do you see any murals? | Yes/No/Cannot Tell |
| 17) Do you see any speed bumps? | Yes/No/Cannot Tell |
| 18) Can you see a bus stop and if so, what kind? | Bus stop with shelter/Bus stop with bench/ Bus top with signage only/ Multiple Types/ No bus stop/ Cannot Tell |
| 19) Are there street lights present? | Yes/No/Cannot Tell |
| 20) Do you see any bike facilities (lanes, bike racks, bike route signs, etc.)? | Yes/No/Cannot Tell |
| 21) Do you see any businesses with Spanish language signs? | Yes/No/Cannot Tell |
| 22) Do you see any businesses with English language signs? | Yes/No/Cannot Tell |
| 23) Do you see any businesses with signs in a language other than Spanish or English? | Yes/No/Cannot Tell |
| 24) Would you enjoy walking here? | Would greatly enjoy/ Would somewhat enjoy/ Would not enjoy/ Would prefer never to walk here/ I never enjoy walking/ Cannot Tell |
| 25) Now consider the oldest image at this location: Is this image appropriate for auditing? | Yes/No/Cannot Tell |
| 26) Consider the oldest image at this location: What month was this image recorded? | Jan/Feb/Mar/Apr/May/Jun/  Jul/Aug/Sep/Oct/Nov or Dec |
| 27) Consider the oldest image at this location: What year was this image recorded? | 2009 or earlier/2010/2011/2012/  2013/2014/2015/2016/2017/  2018/2019 or later |
| 28) Consider the oldest image at this location: Is there vacant or undeveloped land? | Yes/No/Cannot Tell |
| 29) Consider the oldest image at this location: Do you see any windows with bars on them? | Yes/No/Cannot Tell |
| 30) Consider the oldest image at this location: Is there garbage, litter, or broken glass in the street or on the sidewalks? | Yes/No/Cannot Tell |
| 31) Consider the oldest image at this location: Are there empty beer or liquor bottles visible in streets, yards, or alleys? | Yes/No/Cannot Tell |
| 32) Consider the oldest image at this location: Is there graffiti, or evidence of graffiti that has been painted over, on buildings, signs, or walls? | Yes/No/Cannot Tell |
| 33) Consider the oldest image at this location: Are there abandoned cars? | Yes/No/Cannot Tell |
| 34) Consider the oldest image at this location: How many buildings, driveways, or other private infrastructure need non-trivial repairs? | None/One/Two or more/Not applicable/ Cannot Tell |
| 35) Consider the oldest image at this location: Do you see burned out buildings? | Yes/No/Cannot Tell |
| 36) Consider the oldest image at this location: Do you see boarded up or abandoned buildings? | Yes/No/Cannot Tell |
| 37) Consider the oldest image at this location: Is there a sidewalk? | Yes/No/Cannot Tell |
| 38) Consider the oldest image at this location: Is the sidewalk continuous? | Yes/No/Cannot Tell |
| 39) Consider the oldest image at this location: Are there major bumps, cracks, holes, or weeds in the sidewalk? | Yes/No/Cannot Tell |
| 40) Consider the oldest image at this location: Is the sidewalk unobstructed? | Yes/No/Cannot Tell |
| 41) Consider the oldest image at this location: Should there be a sidewalk here? | Yes/No/Cannot Tell |
| 42) Consider the oldest image at this location: Do you see any murals? | Yes/No/Cannot Tell |
| 43) Consider the oldest image at this location: Do you see any speed bumps? | Yes/No/Cannot Tell |
| 44) Consider the oldest image at this location: Can you see a bus stop and if so, what kind? | Bus stop with shelter/Bus stop with bench/ Bus top with signage only/ Multiple Types/ No bus stop/ Cannot Tell |
| 45) Consider the oldest image at this location: Are there street lights present? | Yes/No/Cannot Tell |
| 46) Consider the oldest image at this location: Do you see any bike facilities (lanes, bike racks, bike route signs, etc.)? | Yes/No/Cannot Tell |
| 47) Consider the oldest image at this location: Do you see any businesses with Spanish language signs? | Yes/No/Cannot Tell |
| 48) Consider the oldest image at this location: Do you see any businesses with English language signs? | Yes/No/Cannot Tell |
| 49) Consider the oldest image at this location: Do you see any businesses with signs in a language other than Spanish or English? | Yes/No/Cannot Tell |
| 50) Consider the oldest image at this location: Would you enjoy walking here? | Would greatly enjoy/ Would somewhat enjoy/ Would not enjoy/ Would prefer never to walk here/ I never enjoy walking/ Cannot Tell |
| 51) Now consider both the oldest and newest images: are there signs of public investment such as improved sidewalks or repainted street markings between the oldest and newest? | Yes, a lot/ Yes, a little/ No/ Cannot Tell |
| 52) Now consider both the oldest and newest images: are there signs of private investment such as improved building maintenance between the oldest and newest? | Yes, a lot/ Yes, a little/ No/ Cannot Tell |
| 53) Is there anything else you'd like to say about this location? | (Open text box) |
| *Items used in Item Response Theory model/development of disinvestment score. | |

**Table S2.** Adjusted and fully adjusted incidence rate ratios for different progression of diabetes (primary definition) from baseline to visit 2 based on neighborhood disinvestment z- score at baseline. Comparing adjusted model, which does not contain the NSES index and the fully adjusted model.

|  | **Adjusted^a^** | | | **Fully Adjusted^b^** | | |
| --- | --- | --- | --- | --- | --- | --- |
| **Outcome** | **N** | **Cases at Visit 2** | **IRR (95% CI)** | **N** | **Cases at Visit 2** | **IRR (95% CI)** |
| Primary diabetes (Lab results and/or self-reported medication) | 8360 | 1958 | 0.89 (0.78 – 1.01) | 8346 | 956 | 0.87 (0.77 – 0.99)* |
| Secondary diabetes (Primary + self-reported diagnosis) | 8244 | 1364 | 0.97 (0.88- 1.08) | 8230 | 1362 | 0.95 (0.86 – 1.06) |
| Primary diabetes (Subset of non-movers) | 4380 | 545 | 1.05 (0.92 – 1.18) | 4373 | 544 | 1.05 (0.90 – 1.23) |
| Abbreviations: IRR, incident rate ratio  * P < 0.05  ^a^ Adjusted for age, sex, years living in the US, income, educational attainment, family history of diabetes, and study center/ ethnic heritage  ^b^ Adjusted for age, sex, years living in the US, income, educational attainment, family history of diabetes, NSES, and study center/ ethnic heritage | | | | | | |

**Table S3.** Minimally and fully adjusted incidence rate ratios for different progression of diabetes (primary definition) from baseline to visit 2 based on neighborhood disinvestment z- score at baseline.

|  | **Minimally Adjusted^a^** | | | **Fully Adjusted^b^** | | |
| --- | --- | --- | --- | --- | --- | --- |
| **Progression** | **N** | **Cases at Visit 2** | **IRR (95% CI)** | **N** | **Cases at Visit 2** | **IRR (95% CI)** |
| Pre-diabetes to diabetes | 4565 | 926 | 0.95 (0.87 – 1.04) | 4192 | 854 | 0.91 (0.80 – 1.04) |
| Free of pre-diabetes and diabetes to diabetes | 4555 | 112 | 1.02 (0.79 – 1.33) | 4154 | 102 | 0.66 (0.44 – 0.99)* |
| Free of pre-diabetes and diabetes to either pre-diabetes or diabetes | 4555 | 1888 | 0.99 (0.94 – 1.05) | 4154 | 1744 | 0.95 (0.88 – 1.03) |
| Abbreviations: IRR, incident rate ratio  * P < 0.05  ^a^ Adjusted for age and sex  ^b^ Adjusted for age, sex, years living in the US, income, educational attainment, family history of diabetes, neighborhood SES, and study center/ ethnic heritage | | | | | | |

**Table S4.** Minimally and fully adjusted incidence rate ratios of diabetes (primary definition) at visit 2 for each city based on neighborhood disinvestment z-score at baseline.

|  | **Minimally Adjusted^a^** | | | **Fully Adjusted^b^** | | |
| --- | --- | --- | --- | --- | --- | --- |
| **City** | **N** | **Cases at Visit 2** | **IRR (95% CI)** | **N** | **Cases at Visit 2** | **IRR (95% CI)** |
| The Bronx | 1989 | 239 | 0.87 (0.70 – 1.10) | 1805 | 216 | 0.93 (0.70 – 1.23) |
| Chicago | 2414 | 295 | 0.89 (0.73 – 1.08) | 2261 | 284 | 0.90 (0.74 – 1.09) |
| Miami | 2312 | 225 | 0.87 (0.70 – 1.08) | 1992 | 190 | 0.80 (0.64 – 0.99)* |
| San Diego | 2405 | 279 | 0.91 (0.72 – 1.16) | 2288 | 266 | 0.86 (0.66 – 1.13) |
| Abbreviations: IRR, incident rate ratio  * P < 0.05  ^a^ Adjusted for age and sex  ^b^ Adjusted for age, sex, years living in the US, income, educational attainment, family history of diabetes, neighborhood SES, and ethnic heritage | | | | | | |

**Table S5.** Minimally and fully adjusted incidence rate ratios of diabetes (primary definition) at visit 2 for each city on a subset of participants that did not move during follow-up based on neighborhood disinvestment z-score at baseline.

|  | **Minimally Adjusted^a^** | | | **Fully Adjusted^b^** | | |
| --- | --- | --- | --- | --- | --- | --- |
| **City** | **N** | **Cases at Visit 2** | **IRR (95% CI)** | **N** | **Cases at Visit 2** | **IRR (95% CI)** |
| The Bronx | 1293 | 174 | 0.91 (0.72 – 1.14) | 1190 | 160 | 1.05 (0.78 – 1.42) |
| Chicago | 1176 | 170 | 0.72 (0.52 – 1.00)* | 1099 | 165 | 0.73 (0.56 – 0.96)* |
| Miami | 948 | 86 | 0.86 (0.55 – 1.35) | 833 | 73 | 0.83 (0.52 – 1.31) |
| San Diego | 1310 | 154 | 0.99 (0.78 – 1.26) | 1251 | 146 | 0.94 (0.70 – 1.26) |
| Abbreviations: IRR, incident rate ratio  * P < 0.05  ^a^ Adjusted for age and sex  ^b^ Adjusted for age, sex, years living in the US, income, educational attainment, family history of diabetes, neighborhood SES, and ethnic heritage | | | | | | |
